# Supplementary material for: EnzML: multi-label prediction of enzyme classes using InterPro signatures
Source: BMC Bioinformatics. 2012 Apr 25;13:61. doi: 10.1186/1471-2105-13-61 (PMC3483700; doi:10.1186/1471-2105-13-61)
Supplement: Addtional file 5 — The Java code to format the data files, evaluate and predict. The file enzml_java_code.tar.gz contains the Java code used to format database data to ARFF and XML formats, to execute cross and train-test (jackknife) evaluations and to record evaluation results to database. More information is included in the readme.txt file and the Javadoc files. The code can be used with a MySQL database. To use a different database software, other JDBC drivers might be required. [file 1471-2105-13-61-S5.gz › java_code/utils/doc/uk/ac/ed/inf/utils/RegExpUtils.html]

RegExpUtils


---


|  |  |  |
| --- | --- | --- |
| **Overview** \| **Package** \| **Class** \| **Use** \| **Tree** \| **Deprecated** \| **Index** \| **Help** | |  |
| **PREV CLASS**   **NEXT CLASS** | **FRAMES**    **NO FRAMES**     **All Classes** |  |
| SUMMARY: NESTED \| FIELD \| CONSTR \| METHOD | DETAIL: FIELD \| CONSTR \| METHOD |  |


---


## uk.ac.ed.inf.utils Class RegExpUtils

```
java.lang.Object
  uk.ac.ed.inf.utils.RegExpUtils
```

---

``` public class RegExpUtils extends java.lang.Object ```

---

| **Field Summary** | |
| --- | --- |
| `static boolean` | `m_debug` |


**Constructor Summary**

`RegExpUtils()`


| **Method Summary** | |
| --- | --- |
| `static java.util.Vector<java.lang.String>` | `getAllMatchedSubstring(java.lang.String regExp, java.lang.String text, boolean dotall)`             Returns all regular expression matches in the text (as strings) |
| `static java.util.TreeMap<java.lang.Integer,java.lang.Integer>` | `getAllMatchedSubstringsEndpoints(java.lang.String regExp, java.lang.String text, boolean dotall)`             Gets the start and end index of all regular expression matches in the text |
| `static java.lang.String` | `getFirstMatchedSubstring(java.lang.String regExp, java.lang.String text)`             Gets the first matched substring in the text (dot all mode disabled > the dot does not match the \n character) |
| `static java.lang.String` | `getFirstMatchedSubstring(java.lang.String regExp, java.lang.String text, boolean dotall)`             Gets the first matched substring in the text |
| `static java.util.regex.Matcher` | `getMatcher(java.lang.String regExp, java.lang.String text, boolean dotall)`             Get a matcher trained on the given pattern/regular expression and pointing at the text of interest. |
| `static java.lang.String[]` | `splitTextInLines(java.lang.String text)`             I am splitting the String based on a regular expression which looks for carriage return (\r) or line feed (\n) or carriage return immediately followed by line feed. |
| `static boolean` | `stringMatchesRegExp(java.lang.String string, java.lang.String regExp, boolean dotMatchesNewline)`             matches a string against a regular expression (gives true if there is at least a partial match) |
| `static java.lang.String` | `stripXmlHtmlTags(java.lang.String htmlString)`             Strips all tags from xml or html code |
| `static java.lang.String` | `substituteMatchedString(java.lang.String string, java.lang.String regExp, java.lang.String newString)`             Substitute an old string with a new string if the old string matches the given regular expression |

**Methods inherited from class java.lang.Object**

`equals, getClass, hashCode, notify, notifyAll, toString, wait, wait, wait`

**Field Detail**

### m\_debug

```
public static boolean m_debug
```


**Constructor Detail**

### RegExpUtils

```
public RegExpUtils()
```


**Method Detail**

### getAllMatchedSubstring

```
public static java.util.Vector<java.lang.String> getAllMatchedSubstring(java.lang.String regExp,
                                                                        java.lang.String text,
                                                                        boolean dotall)
```

:   Returns all regular expression matches in the text (as strings)

    :   **Parameters:**: `regExp` -: `text` -: `dotall` - true if the dot should match the newline character too **Returns:**

---


### getAllMatchedSubstringsEndpoints

```
public static java.util.TreeMap<java.lang.Integer,java.lang.Integer> getAllMatchedSubstringsEndpoints(java.lang.String regExp,
                                                                                                      java.lang.String text,
                                                                                                      boolean dotall)
```

:   Gets the start and end index of all regular expression matches in the
    text

    :   **Parameters:**: `regExp` -: `text` -: `dotall` - true if the dot should match the newline character too **Returns:**: a map with key=start of the match, value=end of the match

---


### getFirstMatchedSubstring

```
public static java.lang.String getFirstMatchedSubstring(java.lang.String regExp,
                                                        java.lang.String text)
```

:   Gets the first matched substring in the text (dot all mode disabled > the
    dot does not match the \n character)

    :   **Parameters:**: `regExp` -: `text` - **Returns:**

---


### getFirstMatchedSubstring

```
public static java.lang.String getFirstMatchedSubstring(java.lang.String regExp,
                                                        java.lang.String text,
                                                        boolean dotall)
```

:   Gets the first matched substring in the text

    :   **Parameters:**: `regExp` -: `text` -: `dotall` - if true, it enables dotall mode (the dot matches also a newline) **Returns:**

---


### getMatcher

```
public static java.util.regex.Matcher getMatcher(java.lang.String regExp,
                                                 java.lang.String text,
                                                 boolean dotall)
```

:   Get a matcher trained on the given pattern/regular expression and
    pointing at the text of interest.

    :   **Parameters:**: `regExp` - a regular expression: `text` - the text to be searched for regular expression matches: `dotall` - true if the dot should match the newline character too **Returns:**: a matcher trained on the given regular expression

---


### splitTextInLines

```
public static java.lang.String[] splitTextInLines(java.lang.String text)
```

:   I am splitting the String based on a regular expression which looks for
    carriage return (\r) or line feed (\n) or carriage return immediately
    followed by line feed. These takes care of all types of newlines you may
    encounter. Trailing empty strings are not included in the resulting
    array.

    :   **Parameters:**: `text` - **Returns:**: an array of lines

---


### stringMatchesRegExp

```
public static boolean stringMatchesRegExp(java.lang.String string,
                                          java.lang.String regExp,
                                          boolean dotMatchesNewline)
```

:   matches a string against a regular expression (gives true if there is at
    least a partial match)

    :   **Parameters:**: `string` -: `regExp` -: `dotMatchesNewline` - enables dotall mode (the dot matches also a newline) **Returns:**: true if there is at least a match between the string and the regular expression, false otherwise

---


### stripXmlHtmlTags

```
public static java.lang.String stripXmlHtmlTags(java.lang.String htmlString)
```

:   Strips all tags from xml or html code

    :   **Parameters:**: `htmlString` - **Returns:**: text stripped of tags

---


### substituteMatchedString

```
public static java.lang.String substituteMatchedString(java.lang.String string,
                                                       java.lang.String regExp,
                                                       java.lang.String newString)
```

:   Substitute an old string with a new string if the old string matches the
    given regular expression

    :   **Parameters:**: `string` - the old string: `regExp` - a regular expression: `newString` - the new string to be substituted **Returns:**: 1. null if the string does not match the regular expression, 2. the new string if the old string matches the regexp + the new string is non null/empty, 3. the old string if the old string matches the regexp but the new string is null/empty


---


|  |  |  |
| --- | --- | --- |
| **Overview** \| **Package** \| **Class** \| **Use** \| **Tree** \| **Deprecated** \| **Index** \| **Help** | |  |
| **PREV CLASS**   **NEXT CLASS** | **FRAMES**    **NO FRAMES**     **All Classes** |  |
| SUMMARY: NESTED \| FIELD \| CONSTR \| METHOD | DETAIL: FIELD \| CONSTR \| METHOD |  |


---
